# Temporal precision of regulated gene expression

**DOI:** 10.1371/journal.pcbi.1006201

**Published:** 2018-06-07

**Authors:** Shivam Gupta, Julien Varennes, Hendrik C. Korswagen, Andrew Mugler

**Affiliations:** 1 Department of Physics and Astronomy, Purdue University, West Lafayette, Indiana, United States of America; 2 Hubrecht Institute, Royal Netherlands Academy of Arts and Sciences and University Medical Center Utrecht, Utrecht, the Netherlands; Rice University, UNITED STATES

## Abstract

Important cellular processes such as migration, differentiation, and development often rely on precise timing. Yet, the molecular machinery that regulates timing is inherently noisy. How do cells achieve precise timing with noisy components? We investigate this question using a first-passage-time approach, for an event triggered by a molecule that crosses an abundance threshold and that is regulated by either an accumulating activator or a diminishing repressor. We find that either activation or repression outperforms an unregulated strategy. The optimal regulation corresponds to a nonlinear increase in the amount of the target molecule over time, arises from a tradeoff between minimizing the timing noise of the regulator and that of the target molecule itself, and is robust to additional effects such as bursts and cell division. Our results are in quantitative agreement with the nonlinear increase and low noise of *mig-1* gene expression in migrating neuroblast cells during *Caenorhabditis elegans* development. These findings suggest that dynamic regulation may be a simple and powerful strategy for precise cellular timing.

## Author summary

Cells control important processes with precise timing, even though their underlying molecular machinery is inherently imprecise. In the case of *Caenorhabditis elegans* development, migrating neuroblast cells produce a molecule until a certain abundance is reached, at which time the cells stop moving. Precise timing of this event is critical to *C. elegans* development, and here we investigate how it can be achieved. Specifically, we investigate regulation of the molecule production by either an accumulating activator or a diminishing repressor. Our results are consistent with the nonlinear increase and low noise of gene expression observed in the *C. elegans* cells.

## Introduction

Proper timing is crucial for biological processes, including cell division [1–3], cell differentiation [4], cell migration [5], viral infection [6], embryonic development [7, 8], and cell death [9]. These processes are governed by molecular events inside cells, i.e., production, degradation, and interaction of molecules. Molecular events are subject to unavoidable fluctuations, because molecule numbers are small and reactions occur at random times [10, 11]. Cells combat these fluctuations using networks of regulatory interactions among molecular species. This raises the fundamental question of whether there exist regulatory strategies that maximize the temporal precision of molecular events and, in turn, cellular behaviors.

A canonical mechanism by which a molecular event triggers a cellular behavior is accumulation to a threshold [3, 4, 12–14]: molecules are steadily produced by the cell, and once the molecule number crosses a particular threshold, the behavior is initiated. The temporal precision of the behavior is therefore bounded by the temporal precision of the threshold crossing. Threshold crossing has been shown to underlie cell cycle progression [3] and sporulation [4], although alternative strategies, such as derivative [9] or integral thresholding [15], oscillation [16], and dynamical transitions in the regulatory network [8], have also been investigated.

Recent work has investigated the impact of auto-regulation (i.e., feedback) on the temporal precision of threshold crossing [12, 13]. Interestingly, it was found that auto-regulation generically decreases the temporal precision of threshold crossing, meaning that the optimal strategy is a linear increase of the molecule number over time with no auto-regulation [12] (although auto-regulation can help if there is a large timescale separation and the threshold itself is also subject to optimization [13]). Indeed, even when the molecule also degrades, the optimal precision is achieved when positive auto-regulation counteracts the effect of degradation, preserving the linear increase over time [12]. However, in many biological processes, such as the temporal control of neuroblast migration in *Caenorhabditis elegans* [5], the molecular species governing the behavior increases nonlinearly over time. This suggests that other regulatory interactions beyond auto-regulation may play an important role in determining temporal precision. In particular, the impact of activation and repression on temporal precision, where the activator or repressor has its own stochastic dynamics, remains unclear.

Here we investigate the temporal precision of threshold crossing for a molecule that is regulated by either an accumulating activator or a degrading repressor. Using a first-passage-time approach [12, 17–19] and a combination of computational and analytic methods, we find that, unlike in the case of auto-regulation, the presence of either an activator or a repressor increases the temporal precision beyond that of the unregulated case. Furthermore, the optimal regulatory strategy for either an activator or a repressor corresponds to a nonlinear increase in the regulated molecule number over time. We elucidate the mechanism behind these optimal strategies, which stems from a tradeoff between reducing the noise of the regulator and that of the target molecule, and is similar to the fact that a sequence of time-ordered stochastic events becomes more precisely timed with more events. These findings are robust to more complex features of the regulation process, including bursts of molecule production, more complex regulator dynamics, and cell division. Our results are quantitatively consistent with both the temporal precision and nonlinearity of the *mig-1* mRNA dynamics of the migrating neuroblast cells in *C. elegans* larvae [5]. The agreement of our simple model with these data suggests that many molecular timing processes may benefit from the generic regulatory strategies we identify here.

## Results

**Fig 1:**
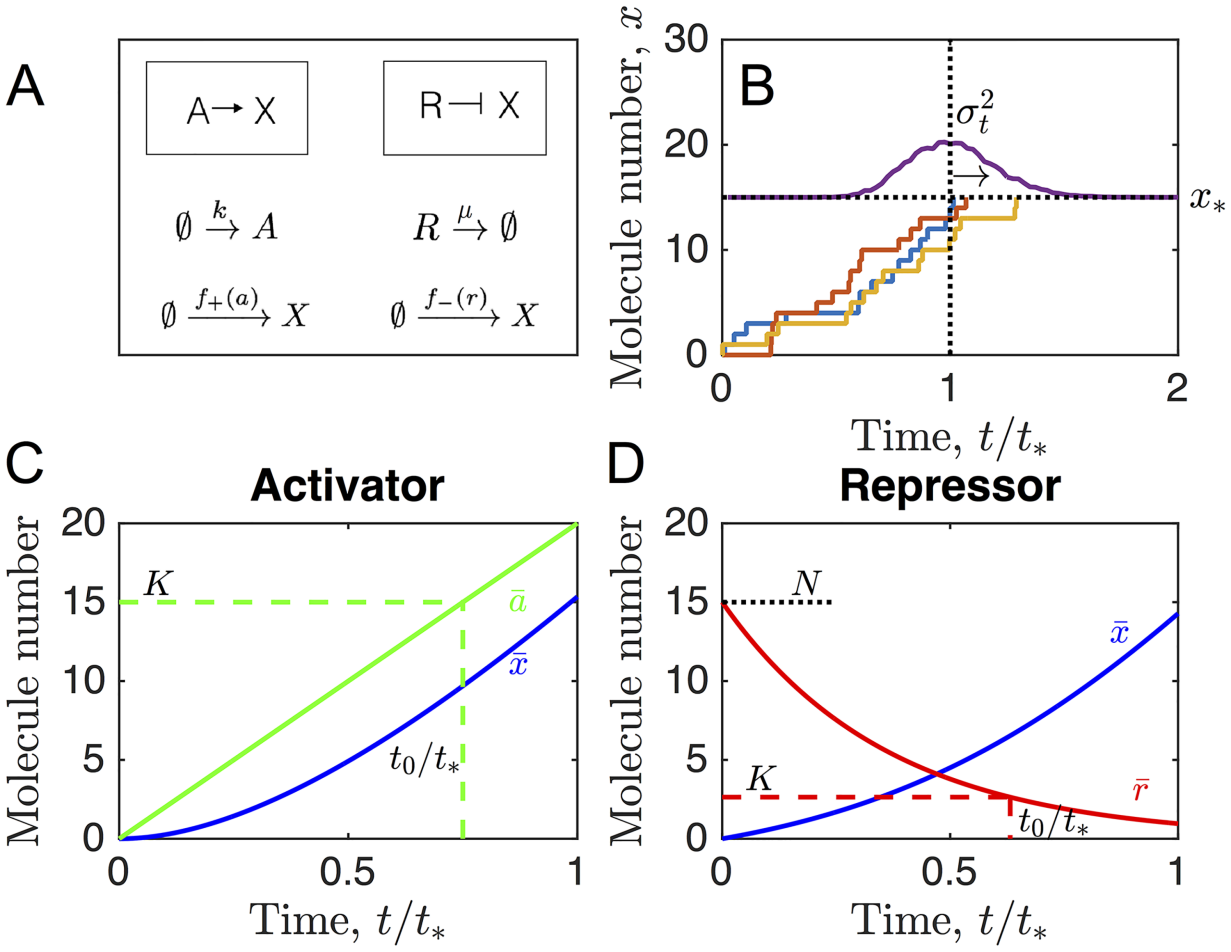
Threshold crossing of a regulated molecular species. (A) A target species *X* is regulated by either an accumulating activator *A* or a degrading repressor *R*. (B) Temporal precision is quantified by the variance $\sigma_{t}^{2}$ of the first-passage time, at which the stochastic molecule number *x* first crosses the threshold x_*_. (C, D) Deterministic dynamics illustrate the effects of regulation. Parameters are kt_*_ = 20 and *K* = 15 in C; μt_*_ = 2.75, *K* = 2.6, and *N* = 15 in B and D; and x_*_ = 15 and *H* = 1 throughout. *t*_0_ is defined by $\bar{a}\left(t_{0}\right)=K$ in C and $\bar{r}\left(t_{0}\right)=K$ in D.

We consider a molecular species *X* whose production is regulated by a second species, either an activator *A* or a repressor *R* (Fig 1A). The regulator undergoes its own dynamics: the activator undergoes pure production at a zeroth-order rate *k* whereas the repressor undergoes pure degradation at a first-order rate *μ*, such that in either case the production rate of *X* increases over time. The activator does not degrade and the repressor is not produced, although we later relax this assumption. For the regulation function we take a Hill function, which is a generic model of cooperative regulation [12, 13, 20],

$$f_{+}\left(a\right)=\frac{\alpha a^{H}}{a^{H}+K^{H}}\;\left(\text{activator}\right),$$ (1)

$$f_{-}\left(r\right)=\frac{\alpha K^{H}}{r^{H}+K^{H}}\;\left(\text{repressor}\right).$$ (2)

Here *a* and *r* are the molecule numbers of *A* and *R*, respectively, *α* is the maximal production rate of *X*, *K* is the half-maximal regulator number, and *H* is the cooperativity. First we neglect additional complexities such as bursts of production, more complex regulator dynamics, cell division, auto-regulation, longer regulatory cascades, or transcriptional delay. Later we check the robustness of our results to bursts, more complex regulator dynamics, and cell division, and we speculate upon the effects of auto-regulation, longer regulatory cascades, and delay in the Discussion.

We suppose that a behavior is initiated when the molecule number *x* crosses a threshold x_*_ (Fig 1B). Because the production of *X* and the dynamics of the regulator are stochastic, the time at which *x* first reaches x_*_ is a random variable. We characterize the precision of this event by the mean $\bar{t}$ and variance $\sigma_{t}^{2}$ of this first-passage time, which we compute numerically from the master equation corresponding to the reactions in Fig 1A (see Materials and methods). The maximal production rate *α* is set to ensure that $\bar{t}$ is equal to a target time t_*_, which we assume is set by functional constraints on the initiated behavior. This leaves *k*, *K*, and *H* as free parameters of the regulation (with *α* a function of these parameters). In principle, these parameters can be optimized to minimize the timing variance $\sigma_{t}^{2}$.

The deterministic dynamics, illustrated in Fig 1C and 1D, neglect fluctuations but give an intuitive picture of the regulation. Whereas the amount of activator increases linearly over time, the amount of repressor decays exponentially from an initial molecule number *N*:

$$\bar{a}\left(t\right)=kt,$$ (3)

$$\bar{r}\left(t\right)=Ne^{-\mu t}.$$ (4)

In either case, the production rate *f*_±_ of *X* increases over time, such that $\bar{x}$ increases nonlinearly. *N* is an additional free parameter in the repressor case.

### Regulation increases temporal precision

To investigate the effects of regulation on temporal precision, we consider the timing variance $\sigma_{t}^{2}$ as a function of the parameters *k* and *K*, or *μ* and *K*. The special case of no regulation corresponds to the limits *k* → ∞ and *K* → 0 in the case of activation, or *μ* → ∞ and *K* → ∞ in the case of repression. In this case, the production of *X* occurs at the constant rate *α*. Reaching the threshold requires x_*_ sequential events, each of which occurs in a time that is exponentially distributed with mean 1/*α*. The total completion time for such a process is given by a gamma distribution with mean $\bar{t}=x_{*}/\alpha$ and variance $\sigma_{t}^{2}=x_{*}/\alpha^{2}$ [19]. Ensuring that $\bar{t}=t_{*}$ requires *α* = x_*_/t_*_, for which the variance satisfies $\sigma_{t}^{2}x_{*}/t_{*}^{2}=1$. This expression gives the timing variance for the unregulated process.

**Fig 2:**
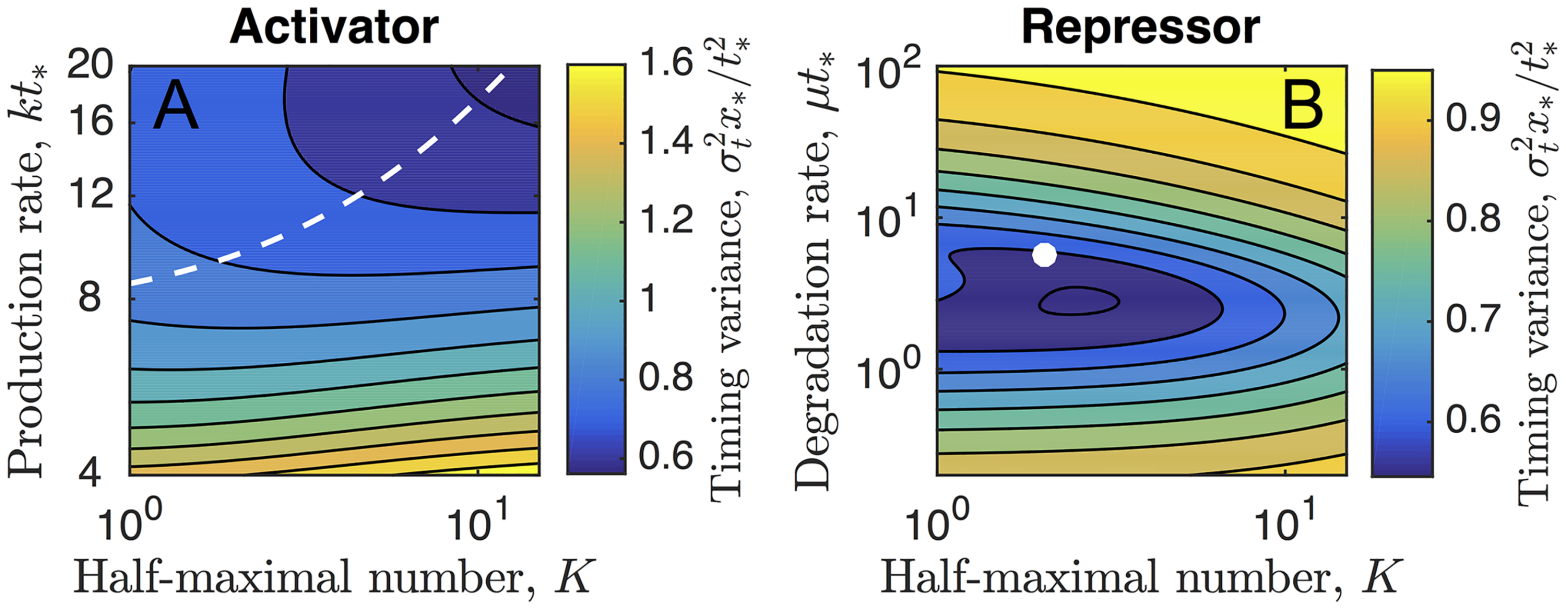
Optimal regulatory strategies. Timing variance as a function of the regulatory parameters reveals (A) a trajectory along which the variance decreases in the case of the activator and (B) a global minimum in the case of the repressor. White dashed line in A and white dot in B show the analytic approximations in Eqs 9 and 11, respectively. Parameters are *N* = 15 in B, and x_*_ = 15 and *H* = 3 in both.

In Fig 2 we plot the scaled variance $\sigma_{t}^{2}x_{*}/t_{*}^{2}$ as a function of the parameters *k* and *K*, or *μ* and *K*, for cooperativity *H* = 3 (color maps). In the case of activation (Fig 2A), the variance decreases with increasing *k* and *K*. This means that the temporal precision is highest for an activator that accumulates quickly and requires a high abundance to produce *X*. In the case of repression (Fig 2B), the variance has a global minimum as a function of *μ* and *K*. This means that the temporal precision is highest for a repressor with a particular well-defined degradation rate and abundance threshold. Importantly, we see that for both activation and repression, the scaled variance can be less than one, meaning that regulation allows improvement of the temporal precision beyond that of the unregulated process. We have checked that this result holds for *H* ≥ 1.

### Optimal regulation balances regulator and target noise

To understand the dependencies in Fig 2, we develop analytic approximations. First, we assume that *H* → ∞, such that the regulation functions in Eqs 1 and 2 become threshold functions. In this limit, the production rate of *X* is zero if *a* < *K* or *r* > *K*, and *α* otherwise. The deterministic dynamics of *X* become piecewise-linear,

$$\bar{x}\left(t\right)=\{\begin{array}{cc} 0 & t<t_{0}\\ \alpha (t-t_{0}) & t\geq t_{0}, \end{array}$$ (5)

where *t*_0_ is determined by either $\bar{a}\left(t_{0}\right)=K$ or $\bar{r}\left(t_{0}\right)=K$ according to Eqs 3 and 4. Then, to set *α*, we use the condition $\bar{x}\left(t_{*}\right)=x_{*}$, which results in *α* = x_*_/(t_*_ − *t*_0_).

Lastly, we approximate the variance in the first-passage time using the variance in the molecule number and the time derivative of the mean dynamics [13]. Specifically, the timing variance of *X* arises from two sources: (i) uncertainty in the time when the regulator crosses its threshold *K*, which determines when the production of the target *X* begins, and (ii) uncertainty in the time when *x* crosses its threshold x_*_, given that production begins at a particular time. The first source is regulator noise, and the second source is target noise. We estimate these timing variances from the associated molecule number variances, propagated via the time derivatives,

$$\sigma_{t}^{2}\approx \underset{\text{regulator}}{\underset{︸}{{\sigma_{y}^{2}{\left(\frac{d\bar{y}}{dt}\right)}^{-2}|}_{t_{0}}}}+\underset{\text{target}}{\underset{︸}{{\sigma_{x}^{2}{\left(\frac{d\bar{x}}{dt}\right)}^{-2}|}_{t_{*}}}},$$ (6)

where *y* ∈ {*a*, *r*} denotes the regulator molecule number.

For the activator, which undergoes a pure production process with rate *k*, the molecule number obeys a Poisson distribution with mean *kt*. Therefore, the molecule number variance at time *t*_0_ is $\sigma_{a}^{2}=kt_{0}$. For the repressor, which undergoes a pure degradation process with rate *μ* starting from *N* molecules, the molecule number obeys a binomial distribution with number of trials *N* and success probability *e*^−*μt*^. Therefore, the molecule number variance at time *t*_0_ is $\sigma_{r}^{2}=Ne^{-\mu t_{0}}\left(1-e^{-\mu t_{0}}\right)$. For the target molecule, which undergoes a pure production process with rate *α* starting at time *t*_0_, the molecule number obeys a Poisson distribution with mean *α*(*t* − *t*_0_). Therefore, the molecule number variance at time t_*_ is $\sigma_{x}^{2}=\alpha \left(t_{*}-t_{0}\right)$. Inserting these expressions into Eq 6, along with the derivatives calculated from Eqs 3–5 and the appropriate expressions for *α* and *t*_0_, we obtain

$$\frac{\sigma_{t}^{2}x_{*}}{t_{*}^{2}}\approx \frac{Kx_{*}}{{\left(kt_{*}\right)}^{2}}+(1-\frac{K}{kt_{*}}{)}^{2}\;\left(\text{activator}\right),$$ (7)

$$\frac{\sigma_{t}^{2}x_{*}}{t_{*}^{2}}\approx \frac{\left(N-K\right)x_{*}}{NK{\left(\mu t_{*}\right)}^{2}}+[1-\frac{\text{log}(N/K)}{\mu t_{*}}{]}^{2}\;\left(\text{repressor}\right).$$ (8)

As a function of kt_*_ and *K*, the global minimum of Eq 7 occurs as kt_*_ → ∞ and *K* → ∞. The path of descent toward this minimum is given by differentiating with respect to *K* at fixed kt_*_ and setting the result to zero, which yields the curve

$$K=\{\begin{array}{cc} 0 & kt_{*}<\frac{x_{*}}{2}\\ kt_{*}-\frac{x_{*}}{2} & kt_{*}\geq \frac{x_{*}}{2}, \end{array}$$ (9)

along which the variance satisfies

$$\frac{\sigma_{t}^{2}x_{*}}{t_{*}^{2}}=\{\begin{array}{cc} 1 & kt_{*}<\frac{x_{*}}{2}\\ \frac{x_{*}}{kt_{*}}(1-\frac{x_{*}}{4kt_{*}}) & kt_{*}\geq \frac{x_{*}}{2}, \end{array}$$ (10)

where the first case comes from the fact that *K* must be nonnegative. In contrast, the global minimum of Eq 8 occurs at finite μt_*_ and *K*: differentiating with respect to each and setting the results to zero gives the values

$$K=e^{-2}N,$$ (11a)

$$\mu t_{*}=\frac{e^{2}x_{*}}{2N}+2,$$ (11b)

$$\frac{\sigma_{t}^{2}x_{*}}{t_{*}^{2}}=\frac{x_{*}}{x_{*}+4e^{-2}N},$$ (12)

where we have assumed that *K*/*N* ≪ 1 (see Materials and methods), which is justified post-hoc by Eq 11a.

These analytic approximations are compared with the numerical results for the activator in Fig 2A (white dashed line, Eq 9) and for the repressor in Fig 2B (white circle, Eq 11). In Fig 2A we see that the global minimum indeed occurs as kt_*_ → ∞ and *K* → ∞, and the predicted curve agrees well with the observed descent. In Fig 2B we see that the predicted global minimum lies very close to the observed global minimum. We have also checked along specific slices in the (*K*, kt_*_) or (*K*, μt_*_) plane and found that the analytic approximations generally differ from the numerical results by about 10% or less, despite the fact that the approximations take *H* → ∞ whereas the numerics in Fig 2 use *H* = 3.

The success of the approximations means that Eq 6 describes the key mechanism leading to the optimal temporal precision. Eq 6 demonstrates that the optimal regulatory strategy arises from a tradeoff between minimizing regulator and target noise. On the one hand, minimizing only the regulator noise would require that the regulator cross its threshold *K* with a steep slope $d\bar{y}/dt$ and therefore at an early time, meaning that the target molecule would be effectively unregulated and would increase linearly over time. On the other hand, minimizing only the target noise would require that the regulator cross its threshold only shortly before the target time t_*_, such that the target molecule would cross its threshold x_*_ with a steep slope $d\bar{x}/dt$, leading to a highly nonlinear increase of the target molecule over time. In actuality, the optimal strategy is somewhere in between, with the regulator crossing its threshold at some intermediate time *t*_0_, and the target molecule exhibiting moderately nonlinear dynamics as in Fig 1C and 1D.

Eqs 10 and 12 demonstrate that the timing variance is small for large kt_*_/x_*_ in the case of activation, and small for large *N*/x_*_ in the case of repression. This makes intuitive sense because each of these quantities scales with the number of regulator molecules: *k* is the production rate of activator molecules, while *N* is the initial number of repressor molecules. To make this intuition quantitative, we define a cost as the time-averaged number of regulator molecules,

$$\left\langle a\right\rangle =\frac{1}{t_{*}}\int_{0}^{t_{*}}dt\;\bar{a}\left(t\right)=\frac{1}{2}kt_{*},$$ (13)

$$\left\langle r\right\rangle =\frac{1}{t_{*}}\int_{0}^{t_{*}}dt\;\bar{r}\left(t\right)=\frac{N}{\mu t_{*}}\left(1-e^{-\mu t_{*}}\right),$$ (14)

where the second steps follow from Eqs 3 and 4. We see that, indeed, 〈*a*〉 scales with *k*, and 〈*r*〉 scales with *N*. Thus, Eqs 10 and 12 demonstrate that increased temporal precision comes at a cost, in terms of the number of regulator molecules that must be produced.

### Model predictions are consistent with neuroblast migration data

We test our model predictions using data from neuroblast cells in *C. elegans* larvae [5]. During *C. elegans* development, particular neuroblast cells migrate from the posterior to the anterior of the larva. It has been shown that the migration terminates not at a particular position, but rather after a particular amount of time, and that the termination time is controlled by a temporal increase in the expression of the *mig-1* gene [5]. Since *mig-1* is known to be subject to regulation [21], we investigate the extent to which the dynamics of *mig-1* can be explained by the predictions of our model.

**Fig 3:**
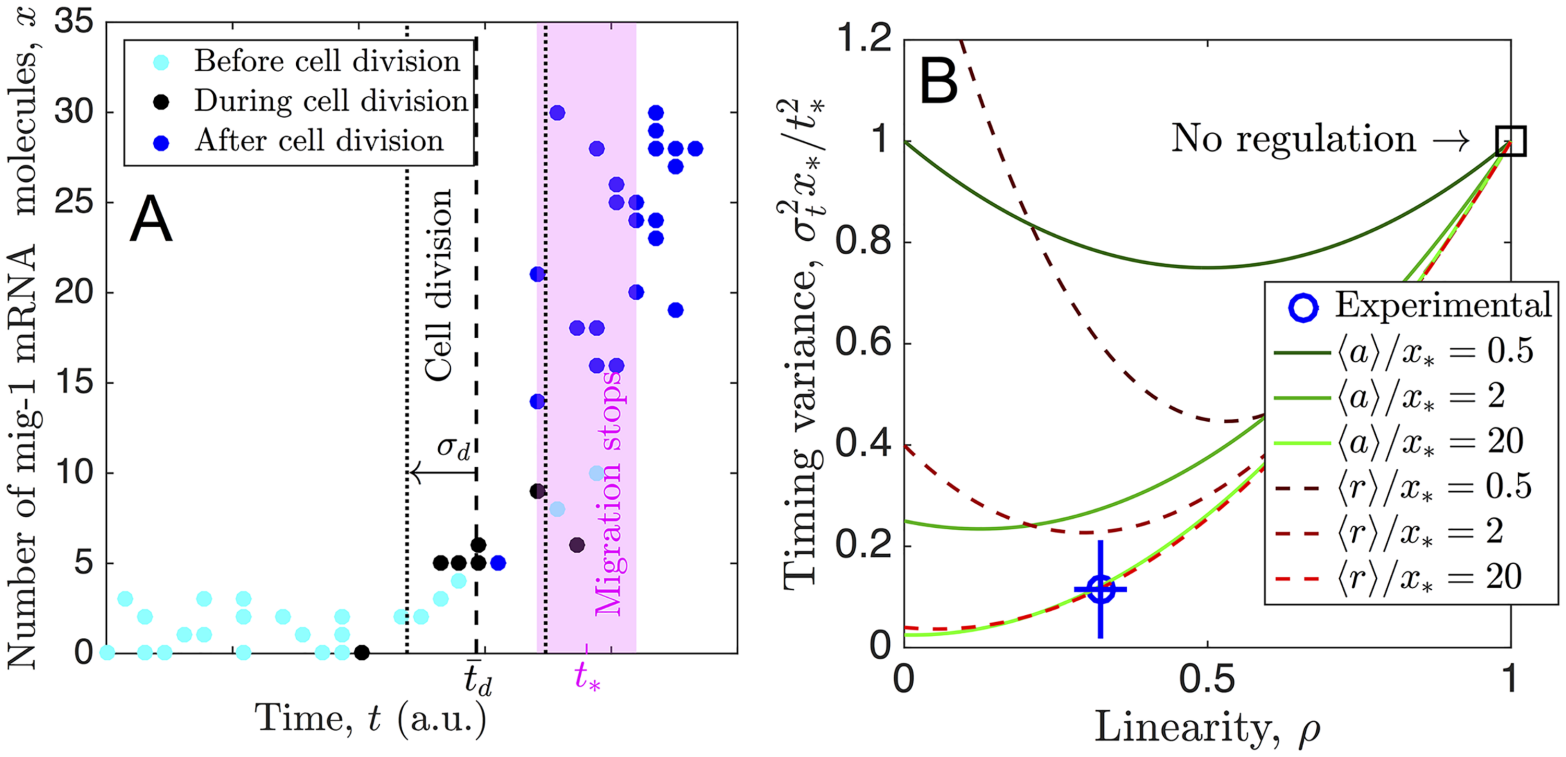
Model predictions agree with neuroblast migration data. (A) Number of *mig-1* mRNA molecules per cell as a function of time *t*, obtained by single-molecule fluorescent in situ hybridization, from [5]. Magenta shows approximate range of times when cell migration terminates. Black lines show mean ${\bar{t}}_{d}$ (dashed) and standard deviation *σ*_*d*_ of cell division times (black points). (B) Timing variance vs. linearity of *x*(*t*), both for experimental data in A (blue circle) and our model (curves, Eqs 16 and 17). Data analyzed using ranges of threshold 10 ≤ x_*_ ≤ 25 and bin size 3 ≤ Δ*x* ≤ 12; error bars show standard deviations of these results. We see that for sufficiently large cost 〈*a*〉/x_*_ or 〈*r*〉/x_*_, model predictions agree with experimental data point.

Fig 3A shows the number *x* of *mig-1* mRNA molecules per cell as a function of time *t*, obtained by single-molecule fluorescent in situ hybridization (from [5]). We analyze these data in the following way (see Materials and methods for details). First, noting that the dynamics are nonlinear, we quantify the linearity using the area under the curve, normalized by that for a perfectly linear trajectory x_*_t_*_/2,

$$\rho =\frac{2}{x_{*}t_{*}}\int_{0}^{t_{*}}dt\;x\left(t\right).$$ (15)

By this definition, *ρ* = 1 for perfectly linear dynamics, and *ρ* → 0 for maximally nonlinear dynamics (a sharp rise at t_*_). Then, we estimate x_*_, t_*_, and the timing variance $\sigma_{t}^{2}$ from the data. Specifically, migration is known to terminate between particular reference cells in the larva [5], which gives an estimated range for the termination time t_*_. This range is shown in magenta in Fig 3A and corresponds to a threshold within the approximate range 10 ≤ x_*_ ≤ 25. Therefore, we divide the *x* axis into bins of size Δ*x*, choose bin midpoints x_*_ within this range, and for each choice compute the mean t_*_ and the variance $\sigma_{t}^{2}$ of the data in that bin. Fig 3B shows the average and standard deviation of results for different values of x_*_ and Δ*x* (blue circle).

The experimental data point in Fig 3B exhibits two clear features: (i) the dynamics are nonlinear (*ρ* is significantly below 1), and (ii) the timing variance is low ($\sigma_{t}^{2}x_{*}/t_{*}^{2}$ is significantly below 1). Neither feature can be explained by a model in which the production of *x* is unregulated, since that would correspond to a linear increase of molecule number over time (*ρ* = 1) and a timing variance that satisfies $\sigma_{t}^{2}x_{*}/t_{*}^{2}=1$ (square in Fig 3B). Furthermore, since auto-regulation has been shown to generically increase timing variance beyond the unregulated case [12], it is unlikely that feature (ii) can be accounted for by a model with auto-regulation alone. Can these data be accounted for by our model with regulation?

To address this question we calculate *ρ* and $\sigma_{t}^{2}x_{*}/t_{*}^{2}$ from our model. For simplicity, we focus on the analytic approximations in Eqs 7 and 8, since they have been validated in Fig 2. In these approximations, since $\bar{x}\left(t\right)$ is piecewise-linear (Eq 5), calculating *ρ* via Eq 15 is straightforward: *ρ* = 1 − *t*_0_/t_*_, where *t*_0_ is once again determined by either $\bar{a}\left(t_{0}\right)=K$ or $\bar{r}\left(t_{0}\right)=K$ according to Eqs 3 and 4. For a given *ρ* and cost 〈*a*〉/x_*_ or 〈*r*〉/x_*_, we calculate the minimum timing variance $\sigma_{t}^{2}x_{*}/t_{*}^{2}$. For the activator, we use the expression for *ρ* along with Eq 13 to write Eq 7 in terms of *ρ* and 〈*a*〉/x_*_,

$$\frac{\sigma_{t}^{2}x_{*}}{t_{*}^{2}}=\frac{x_{*}}{2\langle a\rangle }\left(1-\rho \right)+\rho^{2}.$$ (16)

For the repressor, we use the expression for *ρ* along with Eq 14 to write Eq 8 in terms of *ρ* and 〈*r*〉/x_*_, and then minimize over *N* (see Materials and methods) to obtain

$$\frac{\sigma_{t}^{2}x_{*}}{t_{*}^{2}}=\frac{e^{3}}{27}\frac{x_{*}}{\left\langle r\right\rangle }{\left(1-\rho \right)}^{3}+\rho^{2}.$$ (17)

Eqs 16 and 17 are shown in Fig 3B (green solid and red dashed curves, respectively), and we see the same qualitative features for both cases: all curves satisfy $\sigma_{t}^{2}x_{*}/t_{*}^{2}=1$ at *ρ* = 1, as expected; and as *ρ* decreases, each curve exhibits a minimum whose depth and location depend on cost. Specifically, as cost increases (lighter shades of green or red), the variance decreases, as expected. Importantly, we see that at a cost on the order of 〈*a*〉/x_*_ = 〈*r*〉/x_*_ ∼ 10, the model becomes consistent with the experimental data: both the low timing variance and the low degree of linearity predicted by either the activator or repressor case agree quantitatively with the experiment. This suggests that either an accumulating activator or a degrading repressor is sufficient to account for the temporal precision observed in *mig-1*-controlled neuroblast migration.

### Results are robust to additional complexities including cell division

**Fig 4:**
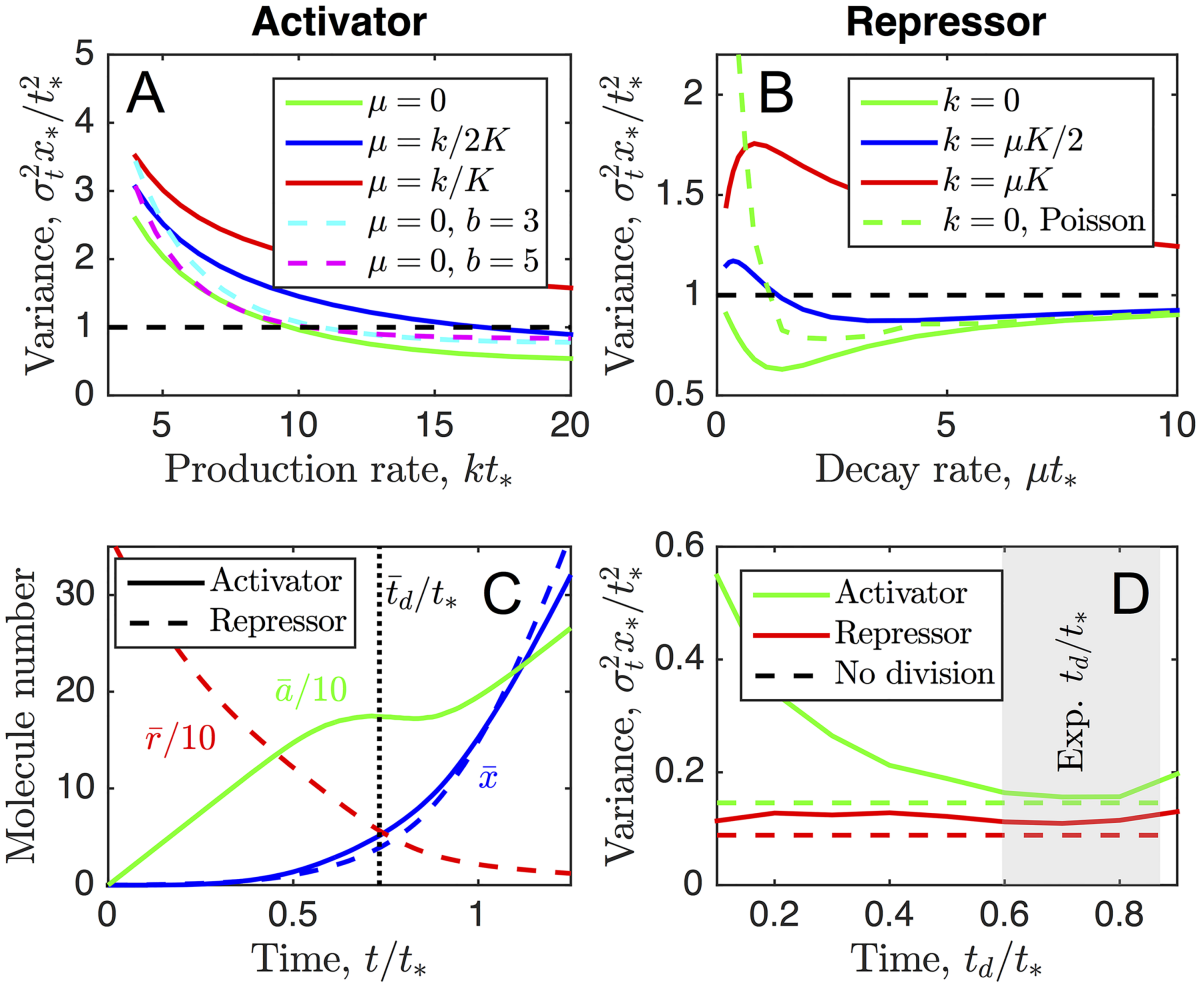
Results are robust to additional complexities including cell division. (A, B) Green solid curves show slices from Fig 2 with *K* = 10 while black dashed line shows unregulated limit $\sigma_{t}^{2}x_{*}/t_{*}^{2}=1$. We see that regulation can reduce timing variance even with bursts in activator production of mean size *b* (A, cyan and magenta dashed), initial Poisson noise in repressor number (B, green dashed), or steady state *k*/*μ* in regulator dynamics (blue) unless it approaches regulation threshold *K* (red). (C) Mean dynamics of activator model (solid) and repressor model (dashed) in which cell division occurs at time ${\bar{t}}_{d}$ on average. Abrupt reductions in molecule numbers are smoothed by noise in *t*_*d*_ and by binomial partitioning of molecules. (D) Timing variance approaches that with no division (dashed) within experimental division region (gray). In A and B, parameters are as in Fig 2. In C and D, parameters are x* = 15, 〈*a*〉/x_*_ = 〈*r*〉/x_*_ = 10, and *H* = 3, with kt_*_, μt_*_, and *K* set to optimal values (Fig 2) and ${\bar{t}}_{d}$ and *σ*_*d*_ set to experimental values. In all cases, *α* is set to ensure that mean threshold crossing time $\bar{t}$ equals t_*_.

Our minimal model neglects common features of gene expression such as bursts in molecule production [22] and additional sources of noise. Therefore we test the robustness of our findings to these effects here. First, we test the robustness of the results to the presence of bursts by replacing the Poisson process governing the activator production with a bursty production process. Specifically, we assume that each production event increases the activator molecule count by an integer in [1, ∞) drawn from a geometric distribution with mean *b* [23, 24]. The limiting case of *b* = 1 recovers the original Poisson process. The results are shown in Fig 4A for *b* = 1, 3, and 5 (green solid, cyan dashed, and magenta dashed curves). We see that bursts in the activator increase the timing variance of the target molecule, as expected, but that there remain parameters for which the variance is less than that for the unregulated case, $\sigma_{t}^{2}x_{*}/t_{*}^{2}=1$ (dashed black line). This result shows that even with bursts, regulation by an accumulating activator is beneficial for timing precision.

We also recognize that whereas the activator can be assumed to start with exactly zero molecules, it is more realistic for the repressor to start with an initial number of molecules that has its own variability. We incorporate this additional variability into the model by performing stochastic simulations [25] of the reactions in Fig 1A and drawing the initial repressor molecule number from a Poisson distribution across simulations. The result is shown by the green dashed curve in Fig 4B. We see that the additional variability gives rise to an increase in the timing variance of the target molecule, as expected (compare with the green solid curve). However, for most of the range of degradation rates, including the optimal degradation rate, the variance remains less than that of the unregulated case, $\sigma_{t}^{2}x_{*}/t_{*}^{2}=1$ (dashed black line). This result indicates that the benefit of repression is robust to this additional source of noise.

Then, we test the robustness of the results to our assumptions that the activator undergoes pure production and the repressor undergoes pure degradation. Specifically, we introduce a degradation rate *μ* for the activator, and a production rate *k* for the repressor, such that either regulator reaches a steady state of *k*/*μ*. The blue curves in Fig 4A and 4B show the case where the increasing activator’s steady state *k*/*μ* is twice its regulation threshold *K*, or the decreasing repressor’s steady state *k*/*μ* is half its regulation threshold *K*, respectively. In both cases, we see that the timing variance of the target molecule increases because the regulator slows down on the approach to its regulation threshold. Nonetheless, we see that it is still possible for the variance to be lower than that of the unregulated case. The red curves show the case where the regulator’s steady state is equal to its regulation threshold. Here we are approaching the regime in which threshold crossing is an exponentially rare event. As a result, the variance further increases, to the point where it is above that of the unregulated case for the full range of parameters shown. These results demonstrate that the benefit of regulation is robust to more complex regulator dynamics, but only if the regulator still crosses its regulation threshold at a reasonable mean velocity.

Finally, we test the robustness of the results to a feature exhibited by the experimental *mig-1* data: near the end of migration, cell division occurs (Fig 3A, black data). One daughter cell continues migrating (Fig 3A, dark blue data), while the other undergoes programmed cell death [5]. To investigate the effects of cell division, we perform stochastic simulations, and at a given time *t*_*d*_ we assume that the cell volume *V* is reduced by a factor of two. For each simulation, *t*_*d*_ is drawn from a Gaussian distribution with mean ${\bar{t}}_{d}$ and variance $\sigma_{d}^{2}$ determined by the data (Fig 3A, black). At *t*_*d*_, we reduce the molecule numbers of both the regulator and the target molecule assuming symmetric partitioning, such that the molecule number after division is drawn from a binomial distribution with total number of trials equal to the molecule number before division and success probability equal to one half. We also reduce the molecule number threshold *K* by a factor of two because it is proportional to the cell volume via *K* = *K*_*d*_*V*, where *K*_*d*_ is the dissociation constant.

Fig 4C shows the dynamics of the mean molecule numbers of the activator (green solid) and its target (blue solid), or the repressor (red dashed) and its target (blue dashed). We see that the activator, repressor, and target drop in molecule number at division but that the abruptness of the drop is smoothed by the variability in the division time. The smoothing is more pronounced in the cases of the repressor and the target because the molecule numbers of these species are smaller at division. Thus, for either the activator or repressor mechanism, we see that the experimentally observed variability in division time is sufficient to smooth out the dynamics of the target molecule number, consistent with the experimental data in Fig 3A.

Additionally, we see in Fig 4D that the timing variance of the target molecule in both the activator and repressor cases is similar to that without division in the region of the experimental division time. This further indicates that either model remains sufficient to account for the low experimental timing variance, even with the experimentally observed cell division. Taken together, the results in Fig 4C and 4D show that the key results of the model are robust to the effects of cell division.

## Discussion

We have demonstrated that regulation by an accumulating activator or a diminishing repressor increases the precision of threshold crossing by a target molecule, beyond the precision achievable with constitutive expression alone. The increase in precision results from a tradeoff between reducing the noise of the regulator and reducing the noise of the target molecule itself. Our minimal model is sufficient to account for both the high degree of nonlinearity and the low degree of noise in the dynamics of *mig-1* in *C. elegans* neuroblasts, providing evidence for the hypothesis that these cells use regulated expression to terminate their migration with increased temporal precision. These results suggest that regulation by a dynamic upstream species is a simple and generic method of increasing the temporal precision of cellular behaviors governed by threshold-crossing events.

Why does regulation increase temporal precision, whereas it has been shown that auto-regulation (feedback) does not [12]? After all, either regulation or positive feedback can produce an acceleration in molecule number over time, leading to a steeper threshold crossing. The reason is likely that positive feedback relies on self-amplification. In addition to amplifying the mean, positive feedback also amplifies the noise. In contrast, regulation by an external species does not involve self-amplification. Therefore, the noise increase is not as strong. The target molecule certainly inherits noise from the regulator (Eq 6), but the increase in noise does not outweigh the benefit of the acceleration, as it does for feedback. Future work could investigate the interplay of regulation and feedback, as well as active degradation of the target molecule, especially as *mig-1* is thought to be subject to feedback and degradation in addition to external regulation [5, 21].

Our finding that regulation increases temporal precision is related to the more basic phenomenon that a sequence of ordered events has a lower relative timing error than a single event [19, 26]. Specifically, if a single event occurs in a time that is exponentially distributed with a mean *τ*, then the relative timing error is *σ*/*τ* = 1. For *n* such events that must occur in sequence, the total completion time follows a gamma distribution with relative timing error $\sigma /\tau =1/\sqrt{n}$, which decreases with increasing *n*. Thus, at a coarse-grained level, the addition of a regulator can be viewed as increasing the length of the sequence of threshold-crossing events from one to two, and thus decreasing the timing error. This perspective suggests that the timing error could be decreased even further via a cascade of regulators.

Although we have demonstrated that our findings are robust to complexities such as bursts and cell division (Fig 4), our model neglects additional features of regulated gene expression such as transcriptional delay. Transcriptional delay has been shown to play an important role in regulation [27, 28] and to have consequences for the mean and variance of threshold-crossing times [29]. If a delay were to arise due to a sequence of stochastic but irreversible steps, then we conjecture that the relative timing error would decrease with the number of these steps, due to the same cascading mechanism mentioned in the previous paragraph. However, it has been shown that if there are reversible steps or cycles within a multistep process, then the first passage time distribution can approach an exponential as the number of steps becomes large [26]. In this case the timing statistics would be captured by our simple model, which assumes single exponentially distributed waiting times. Future work could explore the effects of transcriptional delay in more detail.

Finally, we have shown that the *mig-1* data from migrating neuroblasts in *C. elegans* are quantitatively consistent with either the accumulating activator or diminishing repressor model, but the data do not distinguish between the two models. A direct approach to search for a distinction would be to use genetic knockout techniques to screen directly for regulators of *mig-1* and their effects on its abundance. A less direct approach would be to more closely investigate the effects of the cell division that occurs during migration. For example, we assumed in this study that the volume fraction is equal to the average molecule number fraction in the surviving cell after division. However, if they were found to be unequal for either *mig-1* or its regulator(s), then the concentrations of these species could undergo an abrupt change after division, which may have opposing consequences for the activator vs. the repressor mechanism. Future studies could use these or related approaches to more concretely identify the role of gene regulation in achieving precise timing during cellular processes.

## Materials and methods

### Computation of the first-passage time statistics

We compute the first-passage time statistics $\bar{t}$ and $\sigma_{t}^{2}$ numerically from the master equation following [12], generalized to a two-species system. Specifically, the probability *F*(*t*) that the molecule number crosses the threshold x_*_ at time *t* is equal to the probability *P*_*y*, x_*_−1_(*t*) that there are *y* regulator molecules (where *y* ∈ {*a*, *r*}) and x_*_ − 1 target molecules, and that a production reaction occurs with rate *f*_±_(*y*) to bring the target molecule number up to x_*_. Since this event can occur for any regulator molecule number *y*, we sum over all *y*,

$$F\left(t\right)=\sum_{y=0}^{Y}f_{\pm }\left(y\right)P_{y,x_{*}-1}\left(t\right),$$ (18)

where *Y* = {*a*_max_, *N*}. The repressor has a maximum number of molecules *N*, whereas the activator number is unbounded, and therefore we introduce the numerical cutoff $a_{\text{max}}=kt_{*}+\sqrt{10kt_{*}}$. The probability *P*_*yx*_ evolves in time according to the master equation corresponding to the reactions in Fig 1A,

$${\dot{P}}_{ax}=kP_{a-1,x}+f_{+}\left(a\right)P_{a,x-1}-\left[k+f_{+}\left(a\right)\right]P_{ax},$$ (19a)

$${\dot{P}}_{rx}=\mu \left(r+1\right)P_{r+1,x}+f_{-}\left(r\right)P_{r,x-1}-\left[\mu r+f_{-}\left(r\right)\right]P_{rx}.$$ (19b)

The moments of Eq 18 are

$$\left\langle t^{m}\right\rangle =\sum_{y=0}^{Y}f_{\pm }\left(y\right)\int_{0}^{\infty }dt\;t^{m}P_{y,x_{*}-1}\left(t\right),$$ (20)

where $\bar{t}=\langle t\rangle$ and $\sigma_{t}^{2}=\langle t^{2}\rangle -{\langle t\rangle }^{2}$. Therefore computing $\bar{t}$ and $\sigma_{t}^{2}$ requires solving for the dynamics of *P*_*yx*_.

Because Eq 19 is linear in *P*_*yx*_, it is straightforward to solve by matrix inversion. We rewrite *P*_*yx*_ as a vector by concatenating its columns, ${\vec{P}}^{⊤}=[\left[P_{00},\ldots ,P_{Y0}\right],\ldots ,\left[P_{0,x_{*}-1},\ldots ,P_{Y,x_{*}-1}\right]]$, such that Eq 19 becomes $\dot{\vec{P}}=M\vec{P}$, where

$$M=[\begin{array}{ccccc} M^{\left(1\right)} & & & & \\ M^{\left(2\right)} & M^{\left(1\right)} & & & \\ & M^{\left(2\right)} & M^{\left(1\right)} & & \\ & & \ddots & \ddots & \\ & & & M^{\left(2\right)} & M^{\left(1\right)} \end{array}].$$ (21)

Here, for *i*, *j* ∈ {0, …, *Y*}, the x_*_ − 1 subdiagonal blocks are the diagonal matrix $M_{ij}^{\left(2\right)}=f_{\pm }\left(i\right)\delta_{ij}$, and the x_*_ diagonal blocks are the subdiagonal matrix $M_{ij}^{\left(1\right)}=-\left[k\left(1-\delta_{ia_{\text{max}}}\right)+f_{+}\left(i\right)\right]\delta_{ij}+k\delta_{i-1,j}$ or the superdiagonal matrix $M_{ij}^{\left(1\right)}=-\left[\mu i+f_{-}\left(i\right)\right]\delta_{ij}+\mu \left(i+1\right)\delta_{i+1,j}$ for the activator or repressor case, respectively. The $\delta_{ia_{\text{max}}}$ term prevents activator production beyond *a*_max_ molecules. The final **M**^(1)^ matrix in Eq 21 contains *f*_±_ production terms that are not balanced by equal and opposite terms anywhere in their columns. These terms correspond to the transition from x_*_ − 1 to x_*_ target molecules, for which there is no reverse transition. Therefore, the state with x_*_ target molecules (and any number of regulator molecules) is an absorbing state that is outside the state space of $\vec{P}$ [12]. Consequently, probability leaks over time, and $\vec{P}\left(t\to \infty \right)=\vec{\emptyset }$. Equivalently, the eigenvalues of **M** are negative.

The solution of the dynamics above Eq 21 is $\vec{P}\left(t\right)=\text{exp}\left(Mt\right)\vec{P}\left(0\right)$. Therefore, Eq 20 becomes $\langle t^{m}\rangle ={\vec{V}}^{⊤}[\int_{0}^{\infty }dt\;t^{m}\text{exp}\left(Mt\right)]\vec{P}\left(0\right)$, where ${\vec{V}}^{⊤}$ is a row vector of length x_*_(*Y* + 1) consisting of [*f*_±_(0), …, *f*_±_(*Y*)] preceded by zeros. We solve this equation via integration by parts [12], noting that the boundary terms vanish because the eigenvalues of **M** are negative, to obtain

$$\left\langle t^{m}\right\rangle ={\left(-1\right)}^{m+1}m!{\vec{V}}^{⊤}(M^{-1}{)}^{m+1}\vec{P}\left(0\right).$$ (22)

We see that computing $\bar{t}=\langle t\rangle$ and $\sigma_{t}^{2}=\langle t^{2}\rangle -{\langle t\rangle }^{2}$ requires inverting **M**, which we do numerically in Matlab. We initialize $\vec{P}$ as *P*_*ax*_(0) = *δ*_*a*0_ *δ*_*x*0_ or *P*_*rx*_(0) = *δ*_*rN*_ *δ*_*x*0_ for the activator or repressor case, respectively.

When including cell division, we compute $\bar{t}$ and $\sigma_{t}^{2}$ from 50,000 stochastic simulations [25].

### Deterministic dynamics

The dynamics of the mean regulator and target molecule numbers are obtained by calculating the first moments of Eq 19, $\partial_{t}\bar{y}=\sum_{yx}y{\dot{P}}_{yx}$ and $\partial_{t}\bar{x}=\sum_{yx}x{\dot{P}}_{yx}$, where *y* ∈ {*a*, *r*}. For the regulator we obtain $\partial_{t}\bar{a}=k$ or $\partial_{t}\bar{r}=-\mu \bar{r}$ in the activator or repressor case, respectively, which are solved by Eqs 3 and 4. For the target molecule we obtain $\partial_{t}\bar{x}=\langle f_{\pm }\left(y\right)\rangle$, which is not solvable because *f*_±_ is nonlinear (i.e., the moments do not close). A deterministic analysis conventionally assumes $\langle f_{\pm }\left(y\right)\rangle \approx f_{\pm }\left(\bar{y}\right)$, for which the equation becomes solvable by separation of variables. For example, in the case of *H* = 1, using Eqs 1–4, we obtain

$$\bar{x}\left(t\right)=\{\begin{array}{cc} \alpha t-(\alpha K/k)\text{log}[(kt+K)/K] & \left(\text{activator}\right)\\ \left(\alpha /\mu \right)\text{log}[\left(N+Ke^{\mu t}\right)/\left(N+K\right)] & \left(\text{repressor}\right). \end{array}$$ (23)

Eq 23 is plotted in Fig 1C and 1D.

When including cell division, we compute the mean dynamics from the simulation trajectories (Fig 4C).

### Details of the analytic approximations

To find the global minimum of Eq 8, we differentiate with respect to kt_*_ and *K* and set the results to zero, giving two equations. kt_*_ can be eliminated, leaving one equation for *K*,

$$\frac{1}{2}\text{log}\frac{N}{K}=1-\frac{K}{N}$$ (24)

This equation is transcendental. However, in the limit *K* ≪ *N*, we neglect the last term, which gives Eq 11.

To derive Eq 17, we use

$$\rho =1-\frac{t_{0}}{t_{*}}=1-\frac{\text{log}N/K}{\mu t_{*}}$$ (25)

where the second step follows from $\bar{r}\left(t_{0}\right)=K$ according to Eq 4; and, from Eq 14,

$$\left\langle r\right\rangle =\frac{N}{\mu t_{*}}\left(1-e^{-\mu t_{*}}\right)\approx \frac{N}{\mu t_{*}}$$ (26)

where the second step assumes that the repressor is fast-decaying, μt_*_ ≫ 1. We use Eqs 26 and 25 to eliminate μt_*_ and *K* from Eq 8 in favor of *ρ* and 〈*r*〉,

$$\frac{\sigma_{t}^{2}x_{*}}{t_{*}^{2}}\approx x_{*}(e^{N(1-\rho )/\langle r\rangle }-1)\frac{{\left\langle r\right\rangle }^{2}}{N^{3}}+\rho^{2}.$$ (27)

For nonlinear dynamics (*ρ* < 1) we may safely neglect the −1 in Eq 27. Then, differentiating Eq 27 with respect to *N* and setting the result to zero, we obtain *N* = 3〈*r*〉/(1 − *ρ*). Inserting this result into Eq 27 produces Eq 17.

### Analysis of the experimental data

To estimate the time at which migration terminates in Fig 3A, we refer to [5]. There, the position at which neuroblast migration terminates is measured with respect to seam cells V1 to V6 in the larva (see Fig. 4D in [5]). In particular, in wild type larvae, migration terminates between V2 and the midpoint of V2 and V1. This range corresponds to the magenta region in Fig 3A (see Fig. 4B, upper left panel, in [5]). Under the assumptions of constant migration speed and equal distance between seam cells, the horizontal axis in Fig 3A represents time.

To compute *ρ* for the experimental data in Fig 3A according to Eq 15 we use a trapezoidal sum. For the choices of x_*_ and t_*_ described in the text, this produces the *ρ* values in Fig 3B.
